# Variant of Ascitic Fluid Bacterial Infections in Patients of Liver Cirrhosis

**DOI:** 10.5005/jp-journals-10018-1152

**Published:** 2016-07-09

**Authors:** Jahangir Alam Sarker, Md Shahinul Alam, Mobin Khan, Mamun-Al Mahtab, Mohammad Shahed Ashraf, Faiz Ahmad Khondaker

**Affiliations:** 1 Department of Hepatology, Shaheed Suhrawardy Medical College, Dhaka, Bangladesh; 2 Department of Hepatology, Bangabandhu Shiekh Mujib Medical University, Dhaka, Bangladesh

**Keywords:** Ascitic fluid bacterial infections, Child Pugh C, Cirrhosis, Variant.

## Abstract

**Background and aims:**

Bacterial infections of ascitic fluid of cirrhotic patients are related to high morbidity and mortality. The aim of the study was to determine the variants of ascitic fluid bacterial infection in patients with advanced cirrhosis (Child Pugh Class C).

**Materials and methods:**

We analyzed 35 consecutive cirrhotic patients of Child Pugh Class C with ascites attending the outpatient department (OPD) of Hepatology Department, Bangabandhu Sheikh Mujib Medical University, Dhaka, Bangladesh from January 2008 to December 2009. Clinical and laboratory parameters of these patients were recorded.

**Results:**

Among the total 35 patients, eight patients were symptomatic and 27 patients were asymptomatic. Out of eight symptomatic patients, two had ascitic fluid bacterial infection (25%), whereas six of 27 asymptomatic patients (22.2%) had ascitic fluid bacterial infection.

**Conclusion:**

Bacterial infection should always be considered in patients with ascites with liver cirrhosis irrespective of their symptoms.

**How to cite this article:**

Sarker JA, Alam MS, Khan M, Mahtab MA, Ashraf MS, Khondaker FA. Variant of Ascitic Fluid Bacterial Infections in Patients of Liver Cirrhosis. Euroasian J Hepato-Gastro-enterol 2015;5(2):131-133.

## INTRODUCTION

Ascites is defined as pathological collection of free fluid in peritoneal cavity.^1^ Approximately 50% of patients with compensated liver disease develop ascites during next 10 years^2^ and infection of ascitic fluid is also found in many patients.^3^ Five variants of ascitic fluid infection are described: spontaneous bacterial peritonitis (SBP), culture-negative neutrocytic ascites (CNNA) (probable SBP), secondary bacterial peritonitis, monomicrobial non-neutrocytic bacterascites (MNB), and polymicrobial bacterascites. Among these, SBP and CNNA are more common ascitic fluid infections. A positive ascitic fluid culture for bacteria was considered essential to establish the diagnosis of SBP. However, relying on ascitic fluid culture for diagnosis of SBP has the disadvantages of poor sensitivity and a relatively long time before the results are known. To circumvent the problem, the ascitic fluid white blood count (WBC) and polymorph nuclear neutrophil (PMN) counts have become the standards for making a diagnosis of SBP.^4^ Studies have shown that not only symptomatic patients but a significant number of asymptomatic patients of liver cirrhosis with ascites are infected with bacteria causing ascitic fluid infection as well as urinary tract infection (UTI), biliary or gastrointestinal tract infection bacteremia, pneumonia and others.^5^ Although these factors are highly relevant in clinical practice, almost no valid information about these factors are known in Bangladesh.

## MATERIALS AND METHODS

This observational cross-sectional study included 35 cirrhotic patients with ascites of Child Pugh class C who attended the Department of Hepatology, Bangabandhu Sheikh Mujib Medical University from January 2008 to December 2009. Both symptomatic (temperature >99°F, abdominal pain, tenderness) and asymptomatic (no history of fever, no history of abdominal pain or abdominal tenderness) patients were enrolled in the study. The study protocol was approved by the institutional Review Board of Bangabandhu Sheikh Mujib Medical University. The variants of ascitic fluid infection were defined by the following standard criteria: SBP; neutrophil leukocyte count in the ascitic fluid >250 cells/mm^3^ in conjunction with a positive bacterial culture without any evidence of surgically treatable sources of infections; CNNA; diagnosed when the ascitic fluid culture results were negative, but the neutrophil count was >250 cells/mm^3^. Secondary bacterial peritonitis was diagnosed when the ascitic fluid culture was positive (usually for multiple organisms), the neutrophil count was 250 cells/mm^3^ or greater and intra-abdominal primary source of infection has been identified (e.g. perforated gut, perinephric abscess). Monomicrobial non-neutrocytic bacterial ascites was diagnosed by positive ascitic fluid culture for a single organism, ascitic fluid PMN count lower than 250 cells/mm^3^ and no evidence of intra-abdominal surgically treatable source of infection. Polymicrobial bacterascites was diagnosed when multiple organisms were seen on Gram stain or cultured from the ascitic fluid and the PMN count was lower than 250 cells/mm^3^.

## STATISTICS

Descriptive statistics, the quantitative data were presented as their means ± SD, while categorical or nominal data were expressed in percentage. The t-test was used to compare quantitative data, the Chi-square test used for categorical data and bivariate correlation test were done to find correlation between variables. All analyses were carried out using SPSS software version 16 (SPSS, Inc. Chicago). p-values of less than 0.05 were considered statistically significant.

## RESULTS

Twenty-one (60%) patients were positive for HBsAg positive, two (5.7%) patients had anti-HCV, and 12 patients were non-B/non-C. Hematological profile of the patients revealed that 29 patients had total WBC count <11000/mm^3^ and six patients had total WBC count >11000/mm^3^. Blood neutrophil was >70% neutrophil was among 18 patients and < 70% neutrophil was among 17 patients. Eight patients were symptomatic (22.9%) and 27 patients were asymptomatic (77.1%).

Ascitic fluid characteristics of study subject showed that total ascitic fluid WBC range was 20 to 1200/mm (725.43 ± 2036), ascitic fluid neutrophil was 0 to 1600/mm (204.91 ± 357.74). Among the study subjects eight patients had ascitic fluid neutrophil count >250 cells/mm, which were diagnostic of ascitic fluid bacterial infection. Ascitic fluid total protein was 0.47 to 2.70 (1.2971 ± 0.5607) gm/dl. Three patients had ascitic fluid total protein 1.80 gm/dl, three patients had 1.0 gm/dl, one patient had 1.10 gm/dl and one patient had 2.70 gm/dl. Ascitic fluid albumin was 0.20 to 170 (0.5943 ± 0.3985).

Ascitic fluid culture showed that among 35 study subjects none had culture positive ascitic fluid bacterial infection, though eight patients had ascitic fluid bacterial infection evidenced by ascitic fluid neutrophil count >250 cells/mm. In this study, eight patients had one type of bacterial infection which is CNAA. Out of eight symptomatic patients, two had CNAA, whereas, out of 27 asymptomatic patients, six had CNAA.

## DISCUSSION

The present study also provides some insights about this fact in Bangladesh. Among total study subjects, eight patients were symptomatic (22.9%) and 27 patients were asymptomatic (77.1%). Out of 27 asymptomatic patients six had ascitic fluid bacterial infection (22.22%). On the other hand, two out of eight symptomatic patients (25%) had ascitic fluid bacterial infection (25). Some authors have described this type of infection as a type of spontaneous bacterial peritonitis known as culture negative SBP while others considered it as a different type of ascitic fluid bacterial infection known as CNNA.^6^ The probable cause of culture negativity may be due to fall of ascitic fluid neutrophil count during recovery period. In different study, it has been reported that CNNA is more common than SBP.^7^ Kamani et al (2008), in which it was shown that out of 675 patients 187 (27.7%) patients with ascitic fluid infection.^8^ Among these 187 patients with ascites fluid infection 44 (23.5%) had SBP while 143 (76.4%) had CNNA. The sample size of this study is small. However, it provides a note of caution that considerable numbers of cirrhosis with ascites may have bacterial infection without any visible symptoms at Bangladesh. These patients should be handled carefully before they progress to develop SBP.
